# Identifying Predictors of Psychological Problems Among Adolescents With Congenital Heart Disease for Referral to Psychological Care: A Pilot Study

**DOI:** 10.1016/j.cjcpc.2022.12.001

**Published:** 2022-12-12

**Authors:** Jordan M. Gosnell, Michael T.M. Finn, Darcy N. Marckini, Azizur R. Molla, Heather A. Sowinski

**Affiliations:** a Betz Congenital Heart Center, Helen DeVos Children’s Hospital of Corewell Health, Grand Rapids, Michigan, USA; b Department of Public Health, Grand Valley State University College of Health Professions, Allendale, Michigan, USA; c Department of Pediatrics and Human Development, Michigan State University College of Human Medicine, Grand Rapids, Michigan, USA; d Office of Research and Education, Corewell Health, Grand Rapids, Michigan, USA

## Abstract

**Background:**

The lifelong care of patients with congenital heart disease (CHD) typically begins at a young age, giving paediatric cardiologists a unique perspective on the mental health of their patients. Our aim was to describe and predict reported psychological problems among adolescents with CHD.

**Methods:**

A retrospective review was performed on patients aged 12-17 years who presented to the congenital cardiology clinic during a 1-year timeframe. The presence of psychological problems was collected along with CHD class, clinical history, developmental delay, and patient demographics. We described the prevalence of psychological problems and then, using machine learning algorithms, trained and tested optimal predictive models.

**Results:**

Of the 397 patients who met inclusion criteria, the lifetime prevalence of any reported psychological problem was 35.5%. The most prevalent reported problems were attention-deficit/hyperactivity disorder (18.9%), anxiety (17.6%), and depression (16.1%). Contrary to our expectations, we could not predict the presence or absence of any psychological problem using routine clinical data. Instead, we found multivariate models predicting depression and attention-deficit/hyperactivity disorder with promising accuracy. Prediction of anxiety was less successful.

**Conclusions:**

Approximately 1 of 3 adolescents with CHD presented with the lifetime prevalence of 1 or more psychological problems. Congenital cardiac programmes are in a position of influence to respond to these problems and impact their patients’ mental health as part of a comprehensive care plan. The discovered models using routine clinical data predicted specific psychological problems with varying accuracy. With further validation, these models could become the tools of routine recommendations for referral to psychological care.

## Graphical abstract

**Figure:**
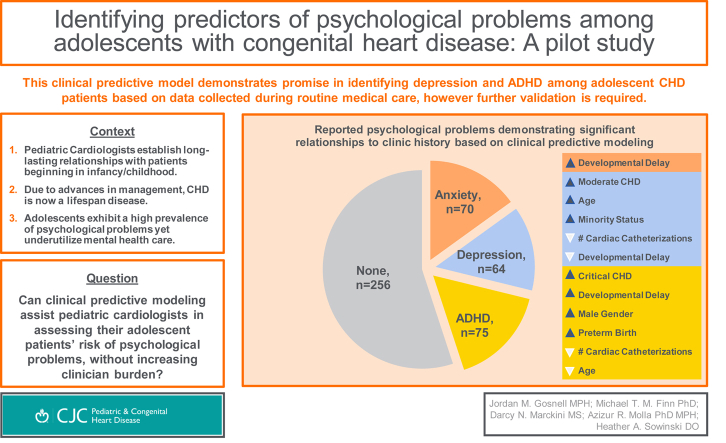

## Résumé

**Contexte:**

Le traitement à vie des patients atteints d’une cardiopathie congénitale commence habituellement dès un jeune âge. Ainsi, les cardiologues pédiatriques ont un point de vue unique sur la santé mentale de leurs patients. Notre objectif était de décrire et de prédire les troubles psychologiques signalés chez les adolescents atteints d’une cardiopathie congénitale.

**Méthodologie:**

Nous avons effectué une revue rétrospective des dossiers de patients âgés de 12 à 17 ans qui se sont présentés dans une clinique de cardiologie pour anomalies congénitales sur une période d’un an. Nous avons recensé toute présence de problèmes psychologiques, en plus de la classe de la cardiopathie congénitale, des antécédents cliniques, des retards de développement et des caractéristiques démographiques des patients. Nous avons également décrit la prévalence des troubles psychologiques; puis, à l’aide d’algorithmes d’apprentissage automatique, nous avons donné une formation sur des modèles prédictifs optimaux et les avons testés.

**Résultats:**

Chez les 397 patients qui avaient répondu aux critères d’inclusion, la prévalence de la survenue d’un problème psychologique au cours de leur vie s’élevait à 35,5 %. Les troubles signalés le plus souvent étaient le trouble déficitaire de l’attention avec hyperactivité (TDAH) (18,9 %), l’anxiété (17,6 %) et la dépression (16,1 %). Contrairement à nos attentes, nous n’avons pas pu prédire la présence ou l’absence de troubles psychologiques à l’aide des données cliniques courantes. Nous avons par contre découvert des modèles multivariés pouvant prédire la dépression et le TDAH avec une précision prometteuse. Toutefois, les prédictions relatives à l’anxiété se sont avérées moins exactes.

**Conclusion:**

La prévalence de la survenue d’au moins un problème psychologique chez les adolescents atteints de cardiopathie congénitale au cours de leur vie était d’environ un adolescent sur trois. Dans le cadre d’un plan de soins complet, les programmes en cardiologie congénitale se trouvent en position d’influence pour répondre à ces problèmes et ainsi améliorer la santé mentale des patients. Par ailleurs, les modèles que nous avons découverts à l’aide des données cliniques courantes ont permis de prédire des problèmes psychologiques en particulier, mais avec une précision variable. Après une validation plus poussée, nous pourrions recommander ces modèles de façon systématique afin d’orienter les patients vers des soins psychologiques.

It is well known that congenital heart disease (CHD) is the most common type of birth defect, with a birth incidence of approximately 1 per 100 births.^1^^,^^2^ Long viewed as a paediatric-specific disease, patients with CHD now regularly survive into adolescence and adulthood as a result of advancements in surgical correction and clinical management.^3^ Now, it is estimated that 2.4 million people in the United States alone are living with CHD, 12% of whom are diagnosed with severe CHD.^4^ This increasing awareness of CHD as a lifespan disease has prompted study into adolescent and adult-onset comorbidities. It is now well known that adolescent and adult patients with CHD are at significant risk for psychological problems that can have a negative effect on physical health and disability.^5^, ^6^, ^7^, ^8^, ^9^, ^10^, ^11^, ^12^, ^13^ In the general population, adolescents exhibit a lifetime prevalence of 49.5% for psychological disorders, and mental health services among adolescents are often underutilized.^13^^,^^14^ Clinical presentation of CHD typically occurs in infancy or childhood and the patient is supported by cardiology specialists beginning at a young age, beginning a patient-clinician relationship that will likely last several years or decades. Given the clinical context of this patient population, along with the adolescent concerns about psychological disorders, there is an opportunity for the paediatric cardiology clinic to be a gateway for psychological care.

The question then becomes: how can paediatric cardiologists be empowered to assess their patients’ risk of psychological problems, allowing for appropriate psychology referrals, without increasing clinician burden? Part of the answer may be found in 2 emerging and synchronistic areas of medicine: clinical predictive models based on machine learning (ML) and precision medicine.^15^ There are various types of ML; however, in the clinical context, ML can be defined as computer systems that can learn, either supervised or unsupervised, using algorithms to analyse and draw inferences from patterns found in patient data for descriptive, predictive, or prescriptive purposes. ML is being explored by many medical specialties to allow clinicians to use the breadth of data available to create patient-specific care plans or to stratify risk in groups of patients with specific characteristics.^16^, ^17^, ^18^, ^19^, ^20^, ^21^, ^22^, ^23^ In paediatric cardiology, the utility of predictive models is being explored with a focus on cardiac surgery, cardiac imaging, interventional procedures, risk stratification, and length of stay, among other topics.^15^^,^^24^, ^25^, ^26^, ^27^, ^28^, ^29^, ^30^ ML is also being effectively explored in the field of psychology, including the risk assessment of specific patient groups.^31^, ^32^, ^33^, ^34^, ^35^, ^36^ Although this is an emergent field of study in paediatric cardiology, there are excellent review articles and editorials explaining ML and its potential value to the cardiovascular field.^37^, ^38^, ^39^, ^40^, ^41^, ^42^, ^43^

The aim of this study is to describe our population from a representative sampling and to work towards the discovery of a pragmatic set of predictors using ML, which could be readily applied to the congenital cardiology clinic. To this end, we stayed “within the chart” in our data analysis, exploring the predictors of chart-noted psychological problems from patient data that would be readily available to the everyday cardiology practice. With sufficient replication, we hope that this line of research can eventually provide a reliable and valid clinical tool for estimating the likelihood of psychological problems in patients with CHD.

## Materials and Methods

A retrospective chart review was performed on patients, 12-17 years of age, who presented to the congenital cardiology clinic of a large tertiary-level medical system located in the Midwestern United States during a 12-month timeframe from 2018 to 2019.

Patients meeting the following criteria were included in the sample population for the chart review: diagnosis of CHD, occurrence of a clinical visit in a 1-year timeframe, and an age of 12-17 years at the time of the clinic visit. Patients were excluded with a documented diagnosis of genetic and/or syndromic abnormalities including but not limited to 1p36 deletion, fetal alcohol syndrome, CHARGE syndrome, cri du chat, Ellis-van Creveld, Holt-Oram, 11q terminal deletion, Marfan, DiGeorge, Noonan, rubella, trisomy 13, trisomy 18, trisomy 21, Turner, VACTERL, Williams, and Wolf-Hirschhorn (additional rare conditions were excluded as applicable). Patients with genetic syndromes were excluded from the sample because of the known associations with developmental delay and are routinely grouped into a different risk category and referred to the neurodevelopmental clinic.

The review was conducted using electronic medical records with covariate extraction from cardiology appointment notes and patient self-report general questionnaires regularly administered during clinic appointments.

The outcome variable is the lifetime presence or absence of a reported psychological problem. The presence of a psychological problem outcome was determined from 3 primary areas within the patient chart: documentation of psychological diagnoses, documentation of psychological problems in cardiology clinic notes, and self-reported patient medical history documentation, which are administered regularly at the beginning of outpatient clinic appointments. These 3 collection methods were used as some patients who seek subspecialty care at the study site receive general care at other institutions. Self-reported patient medical history documents specifically request information regarding the patient’s history of depression, anxiety, counselling or psychological testing, learning problems, and reasons for previous overnight hospital admissions. For the purposes of this study, psychological problems were categorized into anxiety, depression, attention-deficit/hyperactivity disorder (ADHD), bipolar disorder, and other.

Covariates include standard patient demographics of age, sex assigned at birth, and race. To evaluate the association of clinical history to reports of psychological problems, several covariates associated with cardiac clinical history were collected. CHD classification was assigned based on the 2018 American Heart Association (AHA) guidelines—mild, moderate, or severe complexity.^44^ Collected CHD diagnoses were stratified based on these guidelines. Given the comorbidity of development delay with psychological disorders, a diagnosis of developmental delay was also collected; however, specific diagnoses of developmental delay were not captured. Gestational age at birth was also collected because of known comorbid risks with developmental delays and psychological disorders.^45^, ^46^, ^47^

### Predictor selection rationale

The purpose behind the selection of covariates to be used as predictors was two-fold. First, the availability for data to be readily extracted from nonstandardized clinician reports limited how extensive the list of covariates could be. Secondly, the study goal determined that the focus of the analysis was on clinical covariates that could be easily extracted from within the chart. These 2 factors dictated that the clinical predictive model concentrates on cardiac clinical history, preterm history, and standard demographics. These factors also determined, and consequentially limited, how the study captured the reports of psychological problems. Clinical cardiac covariates were a primary focus of this study as it was posited that the severity of CHD lesion along with interventions, surgeries, and the corresponding course of care may influence psychological outcomes. Other aspects of a patient’s history, such as socioeconomic status and sociocultural characteristics, were not included in this pilot study. In addition, externally validated psychological diagnostic evaluations were not available for this sample population. However, given the nature of ML and clinical predictive models, all relevant information of a patient’s history would be expected to be included in a final iteration.

### Analysis

#### Sample size

Based on findings by the National Institutes of Mental Health, there was a 50% predicted prevalence of psychological disorders in the study population.^13^ A sample size calculation using a z-approximation found a necessary sample size of at least 384 for a 0.05 confidence interval. During the study timeframe, 497 unique patients aged 12-17 years with a diagnosis of CHD visited the study site. On completion of the chart review, 100 patients were excluded based on study criteria bringing the final sample to 397 patients.

#### Descriptive statistics and clinical data

Descriptive statistics and univariate analysis were performed, where continuous variables were presented as mean (± standard deviation) or median (interquartile range) depending on the normality of distribution, whereas categorical variables were presented as counts and percentages. Differences between the positive or negative psychological problem outcome groups were analysed using the *t*-test, χ^2^ test, or Fisher’s exact test based on normality.

Variables were re-coded in the following ways: age at first surgery was used to code to a critical CHD exposure variable (cardiac surgery at <1 year of age vs >1 year of age). Because of limitations in the sample size, specific psychological problems were combined to create a binary “presence of any psychological problem” variable. Of note, there were insufficient observations to run models on bipolar and “other” psychological problems in this sample. Because of low numbers of observations in some groups, the race-ethnicity variable was categorized as White, Black, Hispanic, and other. Gestational age, again due to low categorical observation counts, was dichotomized into 2 categories—>36 weeks of gestation and <36 weeks of gestation—to represent the history of full-term birth and preterm birth, respectively.

Contingency tables were used to describe the relationship between psychological problem outcomes and the following variables: CHD classification, history of critical CHD, history of cardiac intervention (surgery, catheterization, or either), total number of cardiac surgeries, age at first surgery (both grouped <1 year, 1-5, 5-10, 10-15, 15-18, and by year), total number of cardiac catheterizations, and neurodevelopment delay diagnosis. An additional contingency table was run to assess the proportion of CHD classifications with diagnosed developmental delay.

#### Machine learning for predictors of psychological problems

We explored the predictors of reported psychological problems using ML techniques: developing models on a random subset of the data (training) and validating against the remaining data (testing). An initial model was run to predict the presence of any reported psychological problem. Then, 3 additional models were created to separately predict the presence of depression, anxiety, and ADHD. For each new model, we randomly sampled 70% of the training dataset to train the ML algorithm.

We used cross-validated logistic LASSO regression for ML, which determines the optimal set of variables. In the interest of discovery, we chose the model that minimized error the most. On the remaining 30% of the data for each model (the testing datasets), the accuracy of each trained model was assessed. This allowed for a practical test of the model that was internal to our dataset, lending some additional confidence to the ability of the discovered models to accurately predict psychological problems.

## Results

After the retrospective chart review, there were 397 unique patients meeting inclusion criteria in the study timeframe. Patients were aged 12-17 years with a mean age of 14.5 years. Males comprised the majority of the sample population (61%, n = 241). Racial demographics of the sample population closely mirrored the regional study population demographics. Complete demographic description can be found in Table 1.

**Table 1:** Descriptive statistics: demographics

Covariates	Lifetime prevalence of reported psychological problems								
		Any psychological problem		Anxiety		Depression		ADHD	
	Totals	No	Yes	No	Yes	No	Yes	No	Yes
	N = 397	256 (64)	141 (36)	327 (82)	70 (18)	333 (84)	64 (16)	322 (81)	75 (19)
Demographics									
Age at time of appointment (y)		14.4 ± 1.6	14.6 ± 1.7	14.4 ± 1.7	14.6 ± 1.7	14.3 ± 1.7	15.1 ± 1.5	14.5 ± 1.7	14.3 ± 1.7
Sex		*P* = 0.25		*P* = 0.22		*P* = 0.97		*P* < 0.01	
Male	241 (61)	150 (59)	91 (65)	203 (62)	38 (54)	202 (61)	39 (61)	183 (57)	58 (77)
Female	156 (39)	106 (41)	50 (35)	124 (38)	32 (46)	131 (39)	25 (39)	139 (43)	17 (23)
Race/ethnicity		*P* = 0.11		*P* = 0.83		*P* = 0.58		*P* = 0.02	
White	297 (73)	191 (75)	106 (75)	242 (74)	55 (79)	249 (75)	48 (75)	238 (74)	59 (78)
Black	25 (6)	13 (5)	12 (9)	22 (7)	3 (4)	23 (7)	2 (3)	16 (5)	9 (12)
Hispanic	31 (10)	26 (10)	6 (4)	27 (8)	5 (7)	27 (8)	5 (8)	30 (9)	2 (3)
Other	43 (11)	26 (10)	17 (12)	36 (11)	7 (10)	34 (10)	9 (14)	38 (12)	5 (7)

Data are presented as n (%).

ADHD, Attention-deficit/hyperactivity disorder.

The lifetime prevalence of any reported psychological problem in our sample population was 35.5% (n = 141), with the most prevalent disorders being anxiety (17.6%; n = 70), depression (16.1%; n = 64), and ADHD (18.9%, n = 75). The proportion of psychological outcomes reported increased with severity of CHD class, with 30% of simple CHD, 36% of moderate CHD, and 40% of the severe CHD groups reporting psychological problems. However, when cross-tabulated across CHD classifications, the differences in the prevalence of any psychological problem were not found to be statistically significant (*P* = 0.42). We found some comorbidities, with 2-disorder comorbidities between ADHD, anxiety, and depression ranging from 3.0% (n = 12) to 4.8% (n = 19) of all patients. A total of 2.8% (n = 11) patients had all 3 problems reported in their chart. Although there was some comorbidity, problems reported in any kind of comorbidity with others amounted to approximately 20% (n = 42) of the total number of psychological problems reported in the data.

The reported lifetime prevalence of depression was over twice as high in moderate CHD and severe CHD (18.6% and 17.0%, respectively) when compared with the simple CHD (7.4%). Differences in reported psychological problems across cardiac clinical history did not demonstrate statistical significance in cross-tabulation. Complete descriptive statistics of clinical history can be found in Tables 2 and 3.

**Table 2:** Descriptive statistics: clinical cardiac history

Covariates	Lifetime prevalence of psychological problems								
	Totals	Any psychological problem		Anxiety		Depression		ADHD	
	N = 397	No, N = 256	Yes, N = 141	No, N = 327	Yes, N = 70	No, N = 333	Yes, N = 64	No, N = 322	Yes, N = 75
CHD classification		*P* = 0.42		*P* = 0.64		*P* = 0.055		*P* = 0.76	
Simple	81	57 (22)	24 (17)	65 (20)	16 (23)	75 (23)	6 (9)	68 (21)	13 (17)
Moderate complexity	263	167 (65)	96 (68)	220 (67)	43 (61)	214 (64)	49 (77)	211 (66)	52 (69)
Severe complexity	53	32 (13)	21 (15)	42 (13)	11 (16)	44 (13)	9 (14)	43 (13)	10 (13)
Critical CHD		*P* = 0.51		*P* = 0.22		*P* = 0.67		*P* = 0.08	
No	306	200 (78)	106 (75)	256 (78)	50 (71)	258 (77)	48 (75)	254 (79)	52 (69)
Yes	91	56 (22)	35 (25)	71 (22)	20 (29)	75 (23)	16 (25)	68 (21)	23 (31)
History of cardiac intervention		*P* = 0.80		*P* = 0.24		*P* = 0.85		*P* = 0.87	
No	219	140 (55)	79 (56)	176 (54)	43 (61)	183 (55)	36 (56)	177 (55)	42 (56)
Yes	178	116 (45)	62 (44)	151 (46)	27 (39)	150 (45)	28 (44)	145 (45)	33 (44)
Cardiac surgical history		*P* = 0.23		*P* = 0.34		*P* = 0.24		*P* = 0.26	
No	219	147 (57)	72 (51)	184 (56)	35 (50)	188 (56)	31 (49)	182 (57)	37 (49)
Yes	178	109 (43)	69 (49)	143 (44)	35 (50)	145 (44)	33 (51)	140 (43)	38 (51)
Grouped age at the time of first cardiac surgery (y), N = 176		*P* = 0.12		*P* = 0.15		*P* = 0.59		*P* = 0.74	
<1	91	56 (52)	35 (51)	71 (50)	20 (57)	75 (52)	16 (49)	68 (49)	23 (61)
1-4	44	31 (29)	13 (19)	40 (28)	4 (11)	38 (27)	6 (18)	37 (27)	7 (18)
5-9	17	11 (11)	6 (9)	14 (10)	3 (9)	13 (9)	4 (12)	14 (10)	3 (8)
10-14	17	6 (6)	11 (16)	12 (9)	5 (14)	12 (8)	5 (15)	13 (9)	4 (11)
15-17	7	3 (3)	4 (6)	4 (3)	3 (9)	5 (4)	2 (6)	6 (4)	1 (3)
Cardiac catheterization history		*P* = 0.82		*P* = 0.20		*P* = 0.52		*P* = 0.27	
No	259	166 (65)	93 (66)	218 (67)	41 (59)	215 (65)	44 (69)	206 (64)	53 (71)
Yes	138	90 (35)	48 (34)	109 (33)	29 (41)	118 (35)	20 (31)	116 (36)	22 (29)

Data are presented as n (%).

ADHD, attention-deficit/hyperactivity disorder; CHD, congenital heart disease.

**Table 3:** Descriptive statistics: noncardiac clinical history

Covariates	Lifetime prevalence of psychological problems								
	Total	Any psychological problem		Anxiety		Depression		ADHD	
	N = 397	No, N = 256	Yes, N = 141	No, N = 327	Yes, N = 70	No, N = 333	Yes, N = 64	No, N = 322	Yes, N = 75
Developmental delay diagnosis		*P* < 0.001		*P* < 0.0001		*P* = 0.43		*P* < 0.001	
No	348	235 (92)	113 (80)	297 (91)	51 (73)	290 (87)	58 (91)	292 (91)	56 (75)
Yes	49	21 (8)	28 (20)	30 (9)	19 (27)	43 (13)	6 (9)	30 (9)	19 (25)
Gestational age (wk)		*P* = 0.076		*P* = 0.35		*P* = 0.23		*P* = 0.017	
≥36	324	216 (91)	108 (85)	273 (90)	51 (84)	275 (90)	49 (84)	269 (91)	55 (81)
<36	40	21 (9)	19 (15)	30 (10)	10 (16)	31 (10)	9 (16)	27 (9)	13 (19)

Data are presented as n (%).

ADHD, attention-deficit/hyperactivity disorder.

The overall prevalence of developmental delay was 12.3% among the sample population. The history of any reported mental disorder diagnoses demonstrated a statistically significant relationship to developmental delay status, with 20% of patients reporting any mental disorder having a history of delay compared with 8% of patients with no psychological problem having a history of delay (*P* < 0.001). Anxiety and ADHD diagnoses were both found to have statistical associations with developmental delay status (*P* < 0.0001 and *P* < 0.001, respectively). More than 25% of reports of anxiety and ADHD had a history of developmental delay. These relationships have been presented in previous literature. How these data and other variables connect to predict the presence of psychological problems remained a question we sought to examine with ML models.

### Machine learning for predictors of psychological problems

#### Presence of any psychological problem

We found no model to predict the presence of any psychological problem that adequately fit the criteria set forth in our cross-validated logistic LASSO regression (Table 1). In our data, a general search for predictors of the presence of any reported psychological problem did not return any models. This diverged from what we expected, which was that there would be general factors indicative of psychopathology in general. We proceeded with attempting ML models for predicting the presence of specific psychological problems.

#### Anxiety

We first explored predictors for the presence of the specific psychological problem of anxiety using the same ML process. We discovered that the optimal model included one predictor: presence of developmental delay. In a logistic regression, those with the indicated presence of developmental delay in their chart were approximately 6 times more likely to also have anxiety mentioned as a problem or as a listed diagnosis (95% confidence interval from 2.78 to 13.21 times more likely), *P* < 0.001 (Table 4).

**Table 4:** Machine learning model: relationship of predictors to specific psychological problem outcomes

	Anxiety			Depression			ADHD		
Predictors	Odds ratios	CI	*P* value	Odds ratios	CI	*P* value	Odds ratios	CI	*P* value
(Intercept)	0.16	0.11-0.22	<0.001	0	0.00-0.05	<0.001	0.72	0.04-13.61	0.828
Developmental delay	6.05	2.78-13.21	<0.001	0.78	0.21-2.27	0.677	2.36	0.99-5.41	0.047
Racial minority				1.64	0.75-3.46	0.204			
Total number of cardiac catheterizations				0.75	0.49-1.07	0.143	0.65	0.40-0.95	0.048
Moderate CHD				2.75	1.06-8.67	0.055			
Complex CHD				6.32	1.61-27.05	0.009			
Age				1.28	1.04-1.59	0.02	0.94	0.77-1.13	0.5
Male sex							0.47	0.23-0.92	0.033
Known preterm birth							1.74	0.67-4.23	0.237
Critical CHD							1.69	0.76-3.69	0.189
Observations	278			278			279		
R2 Tjur	0.088			0.06			0.058		

ADHD, attention-deficit/hyperactivity disorder; CHD, congenital heart disease; CI, confidence interval.

#### Depression

A set of predictors were discovered by the ML process as optimally predictive of the presence of depression listed in patients’ charts. The full model included age as a strong positive predictor, racial/ethnic minority status, fewer cardiac catheterizations, presence of moderate CHD diagnosis (vs simple or complex), and absence of developmental delay (Table 4). Overall, less variance was explained than in the anxiety model.

#### ADHD

Similar to depression, a set of multiple predictors were discovered by the ML procedure and with a similar level of variance explained. The presence of developmental delay, male sex, known preterm birth, fewer cardiac catheterizations, younger age (though a very small effect), and critical CHD predicted the presence of an ADHD-related problem.

### Testing machine-learned models

#### Anxiety

Developmental delay history alone did not perform well as a predictor of anxiety problems in the test data. The area under the curve (AUC) was 0.51. Accuracy was poor at 0.37, where the presence of a delay was somewhat specific for anxiety, 0.76, but not sensitive to it, 0.29.

#### Depression

The discovered model for depression performed better than anxiety, though not highly reliable, with AUC = 0.63 at the optimal cut point (0.18). Accuracy was moderate at 0.64, with balanced sensitivity at 0.63 and specificity at 0.64.

#### ADHD

The model for ADHD performed best of the 3 discovered models at the optimal cut point (0.21). The AUC was 0.73. Accuracy was good at 0.77, though was less sensitive at 0.56 and more specific at 0.82.

## Discussion

This study is an attempt to understand reported psychological problems of adolescent patients with CHD using readily available clinical tools. The main goal is to effect a change in the identification of psychological problems and in referral protocols for adolescent patients in need of mental health services. Of clinical significance, greater than one-third of patients reported 1 or more lifetime psychological problems. This prevalence alone is sufficient to justify using resources to further assess psychological problems in this patient population. Although the prevalence of reported lifetime psychological problems was below the expected 49% for our sample, the observed rates of anxiety, depression, and ADHD in our sample population were higher than the rates reported elsewhere.^48^ These discrepancies may reflect the exploratory nature of the study and the limitations of the retrospective chart review.

Our initial approach in the search for predictors attempted to characterize patients who have at least one of the listed psychological problems: anxiety, depression, or ADHD. This failed to find any predictors. Turning to each psychological problem individually, we discovered unique sets of predictors for each, which at times directly diverged from one another. Depression and ADHD were the most promising in terms of accuracy of prediction with multivariate optimal models. Anxiety was somewhat more difficult to predict in our model; having a developmental delay appeared to indicate some increased likelihood of anxiety, but that characteristic alone incompletely captured all patients with a listed anxiety problem. Developmental delay was present in all models but interestingly diverged in direction; anxiety and ADHD were more common in the presence of developmental delay, whereas depression was less common. We did not have an explanation for this divergence in direction but were confident that it was not a statistical artefact as these problems were all modelled independently.

This study is an exploratory effort to help cardiologists assess their patients’ mental health based on clinical cardiac history. This method requires no specialized examination or questionnaire and uses existing data commonly found in patient charts. In clinics where clinically validated psychological questionnaires are currently administered, a method such as this may augment patient mental health assessment and perhaps encourage intentional patient-centred advocacy towards engaging with psychological care. Multimethod assessments, multiple methods to garner different kinds of information on the same topic, can often increase sensitivity to true positive cases.^35^^,^^36^ The implementation of these chart-based algorithms with patients’ responses to self-report questionnaires could offer important redundancy to clinical care where administration of a self-report measure may be overlooked as well as increase the effectiveness of screening as a line of information on the same topic but different in kind.

### Strengths and limitations

Our study had several strengths. First, the study site is a large tertiary paediatric medical centre and is the only such centre in the region, giving the institution a large geographic referral base. Secondly, the covariates of sex, age, and racial demographics of the study largely reflect the state’s demographics and more specifically the health system’s region.^49^ Third, our methodologies remained “within the chart,” and used widely accepted diagnostic criteria. AHA classification is a nationally recognized system, and the covariates are commonly collected as part of a patient’s clinical history. Because of these strengths, our results have promising validity though would need to be validated in health care systems external to this dataset before confident implementation in congenital heart clinics.

This study also has recognizable limitations, many of which are commonly found among retrospective studies. Foremost of these limitations is the assessment of reported psychological disorders. Reporting on psychological problems is neither a standard of work nor a standardized process in many congenital cardiac clinics. In part, this is due to the novelty of reporting on noncardiac assessment by the congenital cardiologists. Reporting practices during the study timeframe inhibited a comprehensive review of psychological problems. Consequently, this study primarily relied on patient self-reported medical history and cardiologist notes regarding the history of psychological problems. Further, although there was limited comorbidity among reported psychological problems in our sample, future efforts could build upon the modelling approach presented here with multivariate outcome models allowing for the simultaneous modelling of multiple possible psychological problems instead of modelling each as a univariate outcome. It could then become an empirical matter as to the relative advantages of accounting for comorbidities directly within the statistical modelling.

Lastly, unlike CHD, psychological disorders are not reportable conditions and consequently are not tracked with epidemiologic surveillance in the state of Michigan.^50^ Hence, accurate surveillance of these problems is inherently difficult and is compounded in vulnerable populations, such as children. Because of these reasons, this study was required to primarily rely on patient self-report, which is not as strong of a measure as psychological outcomes documented by a psychologist. In addition, as not every patient in the sample was subjected to a standardized psychological evaluation, it is likely that some patients have never had a comprehensive assessment. As a result, there may be both patients with subclinical mental health disorders and patients without access to mental health care who would account for undiagnosed psychological disorders.

### Future directions

Future research of clinical predictive modelling to determine psychological problems in the CHD population should be aware of several considerations. First, because of largely nonstandard assessment and reporting of psychological problems in the cardiology practice, tracking the impact of ML algorithms on referral patterns and diagnosis after psychological diagnostic evaluation should be prioritized. External validation will provide further data to build the clinical predictive models on. Secondly, the nature of ML and clinical predictive modelling invites large datasets to be used for analysis. Thus, a holistic approach to patient history beyond cardiac clinical history to include factors such as socioeconomic status and socio-cultural factors will assuredly improve the model’s predictive outcomes. In addition, this study used the AHA CHD classifications; however, it may be beneficial to increase the granularity of CHD classifications to possibly increase the clinical accuracy of the model as well. To be clear, the model created during this study is not viable for clinical utilization; however, we hope that additional studies may elucidate which aspects of a CHD patient’s history have significant influences on his or her psychological outcomes and at what point in a patient’s course of care he or she should, if necessary, receive a referral for psychological care. Further iterations of this model are required with multisite validation before real-world application to the paediatric cardiologist’s workflow. However, the primary goal of future iterations should continue to be the creation of a pragmatic model that allows for a simple patient referral recommendation in the context of a normal outpatient visit without increasing the burden of work on the provider.

## Conclusions

The CHD population is inherently vulnerable to acquiring several comorbidities with age, including psychological problems. Other research has used ML clinical predictive models to identify novel risk factors for psychological problems in specific patient groups.^31^^,^^32^^,^^35^ Identification of at-risk patients may allow paediatric cardiologists to refer for psychological care earlier and more consistently, possibly pre-empting a significant psychological event. Anxiety, depression, and ADHD are treatable conditions that, without treatment, can worsen and may negatively impact medical status through functional, relational, and physiological pathways. The study’s clinical predictive model demonstrated potential in identifying depression and ADHD among patients with CHD simply based on data collected in the regular course of medical care. The evidence presented in this study gives initial guidance on how to approach mental health assessment as part of a comprehensive CHD care plan. Expanding the clinical predictive algorithm through a multisite study and validating the model with psychological testing could transform comprehensive care for the CHD population.
